# Novel Membrane Designed Polyether Sulfone Filter Reduces Filtration Membrane Obstruction Rate in Drop‐Type With Adjustable Concentrator Cell‐Free and Concentrated Ascites Reinfusion Therapy (DC‐CART)

**DOI:** 10.1111/aor.14932

**Published:** 2024-12-23

**Authors:** Keita Inui, Yosuke Yamada, Daiki Aomura, Kosuke Sonoda, Makoto Harada, Koji Hashimoto, Yuji Kamijo

**Affiliations:** ^1^ Department of Nephrology Shinshu University School of Medicine Matsumoto Nagano Japan

**Keywords:** CART, filter obstruction, polyether sulfone filter

## Abstract

**Background:**

In cell‐free and concentrated ascites reinfusion therapy (CART), filtration membrane obstruction during the ascites processing step is an important clinical problem. A novel membrane designed polyether sulfone filter (n‐PES) was developed to reduce filter membrane obstruction. However, no clinical studies have compared the performance of n‐PES filters with that of conventional filters. Therefore, we aimed to assess whether n‐PES filters reduce membrane obstruction compared to conventional polyethylene (PE) filters during CART ascites processing.

**Methods:**

This was a single‐center, retrospective, observational, controlled cohort study. We compared ascites processing records from the drop‐type with adjustable concentrator CART (DC‐CART) sessions that used n‐PES filters with those that used conventional PE filters. The primary outcome was the occurrence rate of membrane obstruction. Propensity score matching was used to assemble DC‐CART sessions with comparable baseline ascites characteristics.

**Results:**

Among the 173 DC‐CART sessions, 31 sessions using n‐PES filters and 31 using PE filters with similar propensity scores were included in the analysis. The rate of filter membrane obstruction was significantly lower in the n‐PES group than in the PE group (*p* = 0.049). Additionally, the total treatment time was significantly shorter for the n‐PES group (*p* = 0.006). No sessions experienced issues with the processing procedure.

**Conclusion:**

This is the first study to demonstrate that an n‐PES filter reduces the incidence of filter membrane obstruction in clinical CART sessions using human ascites. The n‐PES filter may reduce the burden on the medical staff performing CART.

## Graphical abstract

This study is a single‐center, retrospective, observational, controlled cohort study. Novel membrane designed polyether sulfone filter (n‐PES filter) reduces filtration membrane obstruction rate and toral treatment duration in drop‐type with adjustable concentrator cell‐free and concentrated ascites reinfusion therapy (DC‐CART), potentially easing the burden on medical staff.

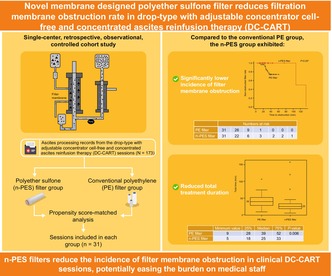

## 1 Introduction

Cell‐free concentrated ascites reinfusion therapy (CART) is a valuable treatment for patients with refractory ascites and is widely used in Japan [1, 2, 3, 4, 5, 6]. In CART, ascites fluid is drained, and cancer cells, other cells, and bacteria in the fluid are removed by filter membranes. The remaining proteins in the fluid are concentrated by removing excess water using a concentrating membrane [1]. The concentrated ascites are then reinfused intravenously into the patient. CART is an established treatment for refractory ascites with various clinical benefits, including preventing protein loss by ascitic fluid removal therapy, improving diuretic resistance, relieving abdominal tension, and improving the quality of life for patients with refractory ascites [1, 7, 8, 9].

Filter membrane obstruction often occurs during ascites filtration in CART, presenting a considerable clinical challenge [10, 11, 12]. To relieve this obstruction, the filtration membranes must be washed, which is time‐consuming and imposes both physical and temporal burdens on the medical staff. Prolonged processing delays the administration of concentrated ascites to patients, leading to intravascular dehydration [7, 13, 14]. Furthermore, membrane washing wastes some medical resources such as wash solution disposal bags, and saline solution [5, 12].

Previously, there was only one type of conventional (PE) filter for CART. However, in recent years, a novel membrane designed polyether sulfone (n‐PES) filter has been developed to reduce filter membrane obstruction [15]. An experimental study evaluating the performance of the n‐PES filter was conducted using bovine blood serum as pseudo‐ascites, revealing that the filter obstruction period was significantly extended compared to using the conventional PE filter [13]. However, no clinical studies have compared the performance of the n‐PES filter with that of conventional filter in real‐world clinical CART sessions using human ascites.

Therefore, this study aimed to determine whether the use of n‐PES filters result in less filter membrane obstruction compared to conventional PE filters during the clinical processing of human ascites for CART.

## 2 Methods

### 2.1 Study Design, Setting, and Participants

**FIGURE 1:**
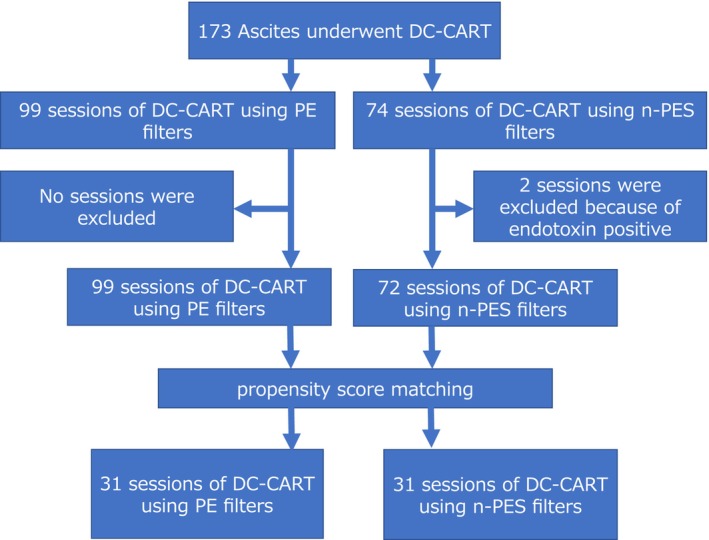
Study selection process. [Color figure can be viewed at wileyonlinelibrary.com].

This was a single‐center, retrospective, controlled cohort study. Ascites processing records from sessions of drop‐type and adjustable concentrator CART (DC‐CART) at Shinshu University Hospital between August 2017 and April 2021 were used. During the study period, 99 DC‐CART sessions were performed using conventional PE filters, while 74 sessions used n‐PES filters (a total of 173 DC‐CART sessions) (Figure 1). All sessions in the PE filter group used conventional PE filters (AHF‐MOW, Asahi Kasei Medical Co., Tokyo, Japan) and polysulfone concentrators (AHF‐UP, Asahi Kasei Medical Co.) (Table 1) [7]. Similarly, all sessions in the n‐PES filter group used n‐PES filters (Mascure filter, SB Kawasumi Co., Tokyo, Japan) and PES concentrators (Mascure concentrator, SB Kawasumi Co.) (Table 1). In the DC‐CART, the filtration process is a drop type with a height differential pressure of 1.7 m, and ascites pass through the filter membrane from the outside to the inside of hollow fibers, while the concentration process uses a roller pump (the detailed process of DC‐CART method has been published [16] and is shown in Information S1). There are no differences in the conditions of the ascites filtration process, including height difference and concentration, for either group. When the filter membrane becomes obstructed, saline is flushed into the system by height differential pressure, and the filter membrane is washed to resume the filtration and concentration process of ascites fluid. The assumed mechanism of filter membrane obstruction and detailed method of filter membrane washing are described in Information S2.

**TABLE 1:** Product specification of filter and concentrator in CART.

	PE filter group	*n*‐PES filter group
Filter		
Name	AHF‐MOW	Mascure filter
Material of hollow fiber	Polyethylene Hydrophilic agent: Ethylene vinyl alcohol copolymer	Polyether sulfone
Inner diameter of hollow fiber	280 μm	250 μm
Thickness of filer	50 μm	75 μm
Maximum trans membrane pressure	66 kPa (500 mmHg)	66.6 kPa (500 mmHg)
Effective filter area	1.5 m^2^	1.3 m^2^
Sterilization method	Gamma sterilization	Gamma sterilization
Fill solution	Sodium chloride solution	Injection solvent
Container size	292 mm × 55 mm	324 mm × 61 mm
Priming volume (inside/outside)	120 mL/270 mL	100 mL/95 mL
Concentrator		
Name	AHF‐UF	Mascure concentrator
Material of hollow fiber	Polysulfone	Polyether sulfone
Inner diameter of hollow fiber	200 μm	160 μm
Thickness of filer	43 μm	30 μm
Maximum trans membrane pressure	66 kPa (500 mmHg)	66.6 kPa (500 mmHg)
Effective filter area	1.5 m^2^	3.0 m^2^
Sterilization method	Gamma sterilization	Gamma sterilization
Fill solution	Sodium pyrosulfite and sodium carbonate solution	Injection solvent
Container size	334 mm × 41 mm	324 mm × 61 mm
Priming volume (inside/outside)	90 mL/140 mL	165 mL/115 mL
Cost (filter+concentrator+circuit)	60 600 yen	63 700 yen

From the 173 DC‐CART sessions, eligible sessions were extracted following specific criteria. The inclusion criteria were as follows: (1) DC‐CART sessions using ascites from patients aged over 20 years at the time of drainage and (2) measurement of the time until the first filter membrane obstruction. Sessions involving ascites detected with endotoxin were excluded. Following these criteria, no sessions were excluded from the PE filter group, while two sessions were excluded from the n‐PES filter group because of endotoxin‐positive ascites. Subsequently, 171 sessions proceeded to the propensity score matching process.

### 2.2 Collection of Baseline Characteristics of Ascites in Patients Undergoing DC‐CART

The following data were collected: volume of ascites, concentration of total protein, albumin, total bilirubin, direct bilirubin, total cholesterol, lactate dehydrogenase, hyaluronic acid, and counts of cells, including monocytes, segmented cells, and other cells. Primary ascitic diseases such as cancer and liver cirrhosis were also recorded.

### 2.3 Outcomes

The primary outcome was the first incidence of filtration membrane obstruction. At our hospital, the time until filter membrane obstruction is documented in the ascites processing reports by clinical medical engineers. This information is used to evaluate the survival rate of filter membranes without obstruction.

The secondary outcomes included the time required to complete the procedure and the number of washes performed in cases of multiple washes. Additionally, the concentration ratio of each substance (total protein, albumin, total bilirubin, direct bilirubin, total cholesterol, lactate dehydrogenase, hyaluronic acid, and cell count in the ascites) was examined. The collection efficiency was determined using the formula: (concentration of substance in reinfused ascites [g/dL] × volume of reinfused ascites [dL])/(concentration of substance in drained ascites [g/dL] × volume of drained ascites [dL]). Moreover, DC‐CART processing problems, including device defects or processing failures that pose clinical problems, were evaluated, and adverse events in patients who underwent DC‐CART were analyzed.

### 2.4 Statistical Analyses

To overcome potential confounders between DC‐CART using n‐PES filters and DC‐CART using PE filters, we performed a 1:1 propensity score matching. Propensity score models were adjusted for the following variables: volume of drained ascites (mL), primary diseases of the ascites, and ascitic laboratory data (total protein, albumin, total bilirubin, direct bilirubin, total cholesterol, lactate dehydrogenase, hyaluronic acid, and number of cells in ascites, including number of monocytes, segmented cells, and other cells). For each analysis, a two‐sided significance level of *p* = 0.05 was used. Quantitative data are expressed as the median (IQR), while qualitative data are expressed as numbers (%). Each parameter was compared between the n‐PES and PE filter groups. In the univariate analysis, the Mann–Whitney *U* test was used for quantitative data, chi‐squared test for qualitative data, and log‐rank test for survival analysis. There were no missing data. Calculations were performed using EZR (Saitama Medical Center, Jichi Medical University, Saitama, Japan) on R Commander version 1.41 (The R Foundation for Statistical Computing, Vienna, Austria) [14].

## 3 Results

### 3.1 Study Population

Of the 171DC‐CART sessions, 62 (31 in the n‐PES filter group and 31 in PE filter groups) were included in the 1:1 propensity score‐matched analysis (Figure 1). Following propensity score matching, all baseline characteristics of ascites were similar between the n‐PES filter and PE filter groups (Table 2; characteristics of the study ascites before propensity score matching are also presented in Table S1). The median ascites volume was 5200 mL in both groups. The most common primary disease was liver cirrhosis.

**TABLE 2:** Characteristics of the study ascites.

	PE filter (*N* = 31)	*n*‐PES filter (*N* = 31)	*p*‐value
Volume of drained ascites (mL)	5200 [3750–7100]	5200 [3500–6000]	0.499
Comorbidities			
Liver cirrhosis	18 (58.1%)	19 (61.3%)	1.000
All cancer	26 (83.9%)	24 (77.4%)	0.749
Gastric cancer	5 (16.1%)	2 (6.5%)	0.425
Liver cancer	9 (29.0%)	7 (22.6%)	0.772
Bile duct cancer	2 (6.5%)	3 (9.7%)	1.000
Ovarian cancer	2 (6.5%)	2 (6.5%)	1.000
Pancreatic cancer	3 (9.7%)	3 (9.7%)	1.000
Other cancer	5 (16.1%)	7 (22.6%)	0.749
Laboratory data			
Ascitic total protein (g/dL)	1.20 [1.00–2.35]	1.70 [0.95–2.40]	0.893
Ascitic albumin (g/dL)	0.60 [0.45–1.20]	0.80 [0.45–1.10]	0.966
Ascitic total bilirubin (mg/dL)	0.45 [0.27–0.60]	0.26 [0.23–0.52]	0.139
Ascitic direct bilirubin (mg/dL)	0.18 [0.10–0.25]	0.12 [0.09–0.20]	0.398
Ascitic total cholesterol (mg/dL)	24 [11–39]	23 [12–37]	0.784
Ascitic lactate dehydrogenase (U/L)	69 [49–307]	58 [40–154]	0.275
Ascitic hyaluronic acid (ng/mL)	4490 [2825–8490]	5200 [3580–7860]	0.588
Number of cells in ascites (/μL)	103 [66–329]	116 [54–245]	0.944
Number of monocyte in ascites (/μL)	66 [31–161]	97 [47–193]	0.426
Number of segmented cell in ascites (/μL)	3 [2–52]	6 [3–12]	0.927
Number of other cells in ascites (/μL)	5 [1–30]	3 [2–7]	0.423

*Note:* Qualitative data, number (%); *p*‐values were calculated by the chi‐squared test. quantitative data, median (IQR); *p*‐values were calculated by the Mann–Whitney *U* test.

### 3.2 Primary Outcome

**FIGURE 2:**
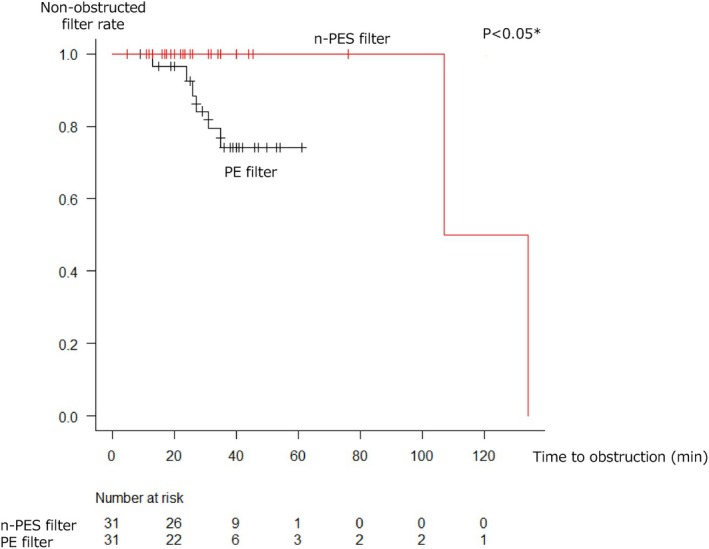
Kaplan–Meier comparison of the time to first filter obstruction. *Filter obstruction rate was compared between the two groups by the log‐rank test. [Color figure can be viewed at wileyonlinelibrary.com].

Among the 62 sessions included in the analysis, at least one filter obstruction occurred in eight sessions, with a median observation time of 27 min (20–40 min). The Kaplan–Meier curves illustrate the non‐obstructive filter survival rate for each group (Figure 2). The n‐PES filter group exhibited a significantly lower incidence of membrane obstruction than the conventional PE filter group (obstruction rate: 2.0 vs. 5.9 per 1000 session minutes, *p* = 0.049). Even when comparing ascites with cancer, the n‐PES filter group had a significantly lower incidence of membrane obstruction than the conventional PE filter group. However, when comparing ascites with liver cirrhosis, the difference was not significant (Figures S1 and S2).

### 3.3 Secondary Outcomes

**FIGURE 3:**
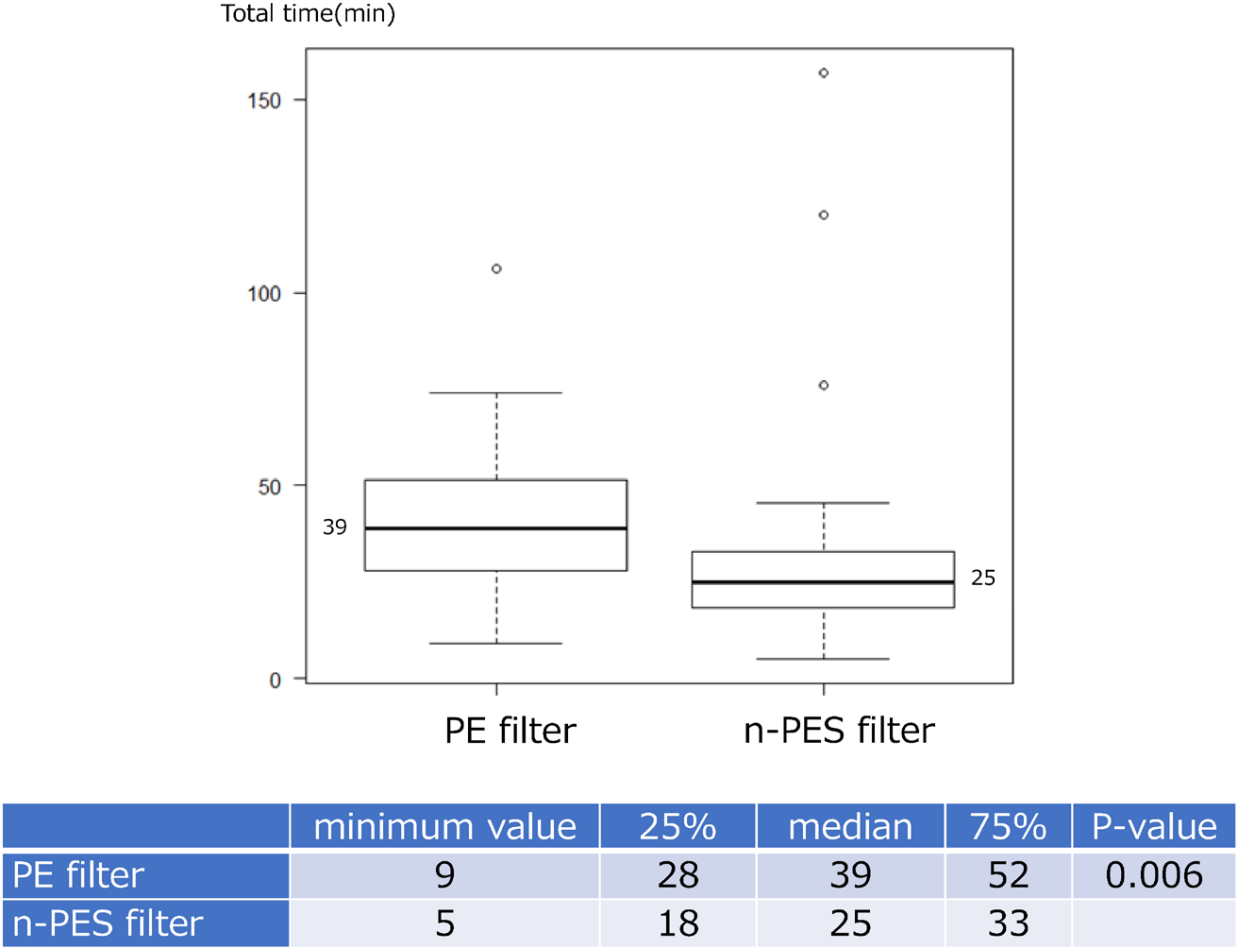
Total time from the start to the end of processing. P‐values were calculated using the Mann–Whitney *U* test. [Color figure can be viewed at wileyonlinelibrary.com].

The time required to complete the procedure was significantly shorter in the n‐PES filter group (Figure 3). The median time was 25 min (18–33 min) in the n‐PES filter group and 39 min (28–51 min) in the PE filter group (*p* = 0.006).

**FIGURE 4:**
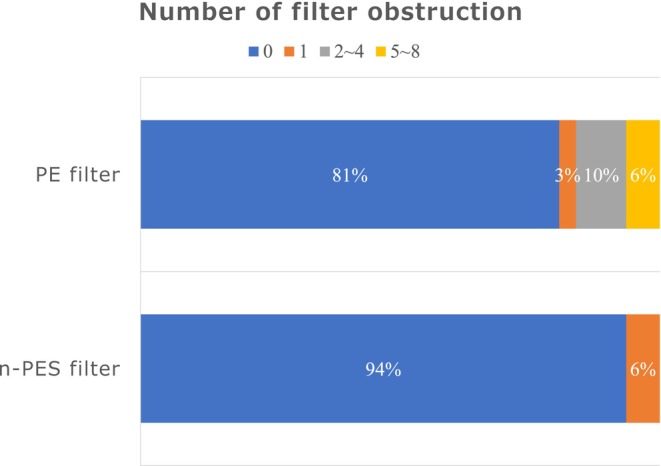
Number of filter membrane obstructions. Blue bar, no filter obstruction group; orange bar, one‐time filter obstruction group; gray bar, 2–4 times obstruction group; yellow bar, 5–8 times filter obstruction group. [Color figure can be viewed at wileyonlinelibrary.com].

Figure 4 shows the number of filter obstructions. In the n‐PES filter group, no cases of obstruction occurred more than once, with 6% (*n* = 2) of cases experiencing one‐time obstruction. In the PE filter group, the incidence of one‐time obstruction was 3% (one case). The incidence of 2–4 obstructions was 10% (2 times: no cases; 3 times: one case; 4 times: two cases) and that of 5–8 obstructions was 6% (5 times: one case; 6 times: no case; 7 times: no case; 8 times: one case).

Table 3 presents the collection efficiencies of each substance. No significant difference was observed in the total protein content (*p* = 0.863). However, the n‐PES filter group had significantly higher collection efficiencies for total cholesterol (*p* < 0.001) and hyaluronic acid (*p* < 0.001) levels and a lower efficiency for direct bilirubin (*p* < 0.001). Cell collection efficiency was 0% in both groups as all cells in the ascites were removed during ascites processing. Additionally, neither group reported any processing problems.

**TABLE 3:** Recovery rate of substances in ascites.^a^

	PE filter	*n*‐PES filter	*p*‐value
*N*	31	31	
Total protein collection	72% [60–77]	68% [58–76]	0.863
Albumin collection	76% [65–84]	68% [55–79]	0.232
Total bilirubin collection	80% [69–85]	73% [59–79]	0.185
Direct bilirubin collection	73% [63–83]	52% [46–60]	< 0.001
Lactate dehydrogenase collection	77% [65–84]	76% [63–80]	0.970
Total cholesterol collection	33% [19–46]	64% [54–70]	< 0.001
Hyaluronic acid collection	18% [6–33]	48% [36–58]	< 0.001
Cell collection	0%	0%	

^a^ Recovery rate = Amount of substance in ascites after process/Amount of substance in ascites before process.

Adverse events that occurred in these patients are detailed in Table S2. No significant differences were observed between the two membranes.

## 4 Discussion

This controlled cohort study showed, for the first time, that n‐PES filters inhibit the occurrence of membrane obstruction in real‐world clinical DC‐CART sessions using human ascites. Other advantages of the n‐PES filter over the PE filter include a reduction in total treatment time and a decrease in the number of membrane obstructions.

Adopting the n‐PES filter in clinical practice can provide many benefits by reducing filter membrane obstruction during DC‐CART. First, using n‐PES filters will reduce the time spent by medical staff in processing CART ascites, which could improve labor shortages in busy clinical settings. Moreover, the n‐PES filter reduced the number of membrane‐washing procedures required, as demonstrated in this study. Second, n‐PES filters can help lower medical costs by reducing the excessive use of medical materials such as wash solution disposal bags and saline solution required for the filtration membrane washing process [12]. Third, using n‐PES filters could shorten the time from ascites drainage to administration, potentially preventing hypoalbuminemia and stabilizing the patient's circulation. Because a large amount of ascites drainage without reinfusion can cause hypoalbuminemia and hypotension due to circulatory deterioration, rapid processing, and reinfusion of ascites are crucial in CART [7, 17, 18, 19]. In this study, the n‐PES filter reduced the total processing time by a median of 14 min compared to the conventional filter. The difference in filter survival rates may have been more pronounced for ascites attributed to cancer, which is more susceptible to obstruction than ascites attributed to cirrhosis. This suggests that the n‐PES filter was particularly effective for ascites associated with malignancy. For these reasons, we believe that n‐PES filters should be used for all DC‐CART to shorten ascites processing time and reduce the burden on technicians, especially when treating ascites with high protein and cellular contents that lead to membrane obstruction. However, the use of n‐PES filters in other types of CART should be considered carefully. Currently, there is a drop‐type CART using inside‐to‐outside filtration. Since we demonstrated the effectiveness of the n‐PES filter only in DC‐CART using outside‐to‐inside filtration, its effectiveness may not be generalized to the drop‐type CART using inside‐to‐outside filtration. Moreover, there are some types of specialized CART machines equipped with multiple roller pumps and an automatic ascites treatment function, including a membrane wash function. There is one type of CART‐specific machine that uses the n‐PES filter as the default, but others are designed to use the conventional PE filter. In these CART machines, the ascites treatment automatic conditions are preset using a conventional PE filter. Using n‐PES filters with the PE filter default‐CART machines can cause various problems including insufficient filtration/concentration and reduced efficiency in protein collection. When using n‐PES filters with these CART machines, the treatment conditions of the CART machine must be adjusted, and their effectiveness and safety must be verified before clinical application. If these verifications are difficult, it is important to select the default CART membrane for each specialized CART machine.

The mechanism by which the n‐PES filter inhibits filter obstruction remains unknown, as Kawasumi Co., the manufacturer, has not disclosed this information owing to patent issues. In addition to the hollow fiber material, there are differences between the two filters, such as the inner diameter and thickness of the fiber, maximum transmembrane pressure, effective filter area, container size, and priming volume (Table 1). Furthermore, the hollow fibers of this n‐PES filter have an asymmetric membrane structure, which is superior to symmetrical membrane structures in terms of solute permeability and clogging prevention. These differences may affect the function of n‐PES filter to prevent membrane obstruction. Further studies are anticipated to elucidate this mechanism.

As mentioned above, several types of dedicated CART machines equipped with an automatic membrane wash function are used clinically. When these specialized CART machines are used, the problem of membrane obstruction may be solved, regardless of the type of filtration membrane used. However, these specialized CART machines are expensive, and not all medical institutions have them. Consequently, these institutions resort to performing drop‐type CART, which does not require specialized CART machines. In such cases, the n‐PES filter may be particularly valuable for institutions that perform drop‐type CART using outside‐to‐inside filtration.

The recovery rates differed for each substance. Although no significant difference was observed in total protein and albumin collection between the two filters, the n‐PES filter had a significantly higher total cholesterol and hyaluronic acid collection. This suggests that cholesterol and hyaluronic acid may pass through the n‐PES filter without adhering to it, resulting in less membrane obstruction. In contrast, the n‐PES filter had a significantly lower direct bilirubin collection, suggesting that factors beyond the molecular weight, such as polarity, may also influence the recovery rate.

Despite the varying recovery rates, no severe adverse events were recorded in either group, indicating that both membranes could safely perform CART [16, 20]. The clinical significance of these different recovery rates remains unknown, emphasizing the need for further studies.

This study had several limitations. First, it was a single‐center study that utilized DC‐CART; therefore, the effectiveness of n‐PES filter in drop‐type CART using inside to outside filtration requires further validation. Second, being a retrospective observational study, other unmeasured factors or outcomes could not be considered. For example, fibrin is considered an important factor for membrane clogging. However, we did not routinely measure fibrin level in ascites, and therefore could not include fibrin levels as a variable in propensity score matching. The first unmeasurable reason is that fibrin clots have often already formed in ascites with a high fibrin content, and the measurable fibrin was prone to be low and did not reflect the total fibrin level in the ascites. The second unmeasurable reason is that fibrin level could not be measured without using spits with sodium citrate, and those spits were not in general use at the time of ascites collection. Therefore, we were unable to evaluate the fibrin levels in the ascites of both groups, and we cannot exclude the possibility that this difference may have influenced the results.

In conclusion, this retrospective controlled study comparing n‐PES and PE filters demonstrates that n‐PES filters reduce the incidence of filtration membrane obstruction in human ascites. Adopting n‐PES filters may help reduce the burden of processing on medical staff and enhance the quality of CART procedures. Future studies should conduct multicenter studies to validate the findings across different clinical settings and populations, ensuring the generalizability of our results.

## Author Contributions

K.I. and Y.Y. designed this study. K.I. and Y.Y. collected the data. K.I. performed the statistical analyses and wrote the draft of the manuscript. Y.Y., D.A., K.S., M.H., K.H., and Y.K. edited the manuscript. All the authors have read the manuscript and approved its publication.

## Ethics Statement

This study was performed in accordance with the tenets of the Declaration of Helsinki and approved by the ethics committee of Shinshu University Hospital (Registration number: 190408). Written informed consent was waived for this study due to its retrospective nature, as it used medical records without subjecting patients to new interventions. Furthermore, as an alternative to written informed consent, an opt‐out document was created and posted on the hospital website. This document contained information about the research design and the publication of results, providing patients the opportunity to withdraw their medical data. The collected data were anonymized and analyzed.

## Conflicts of Interest

The authors declare no conflicts of interest.

## Supporting information


Data S1.

## Data Availability

The datasets generated and analyzed in the current study are available from the corresponding author upon reasonable request.
