# Supplementary material for: Novel Membrane Designed Polyether Sulfone Filter Reduces Filtration Membrane Obstruction Rate in Drop‐Type With Adjustable Concentrator Cell‐Free and Concentrated Ascites Reinfusion Therapy (DC‐CART)
Source: Artif Organs. 2024 Dec 23;49(4):592–9. doi: 10.1111/aor.14932 (PMC11974482; doi:10.1111/aor.14932)

**Novel membrane designed polyether sulfone filter reduces filtration membrane obstruction rate in drop type with adjustable concentrator cell-free and concentrated ascites reinfusion therapy (DC-CART)**

Keita Inui, Yosuke Yamada, Daiki Aomura, Kosuke Sonoda, Makoto Harada, Koji Hashimoto and Yuji Kamijo

**The supplementary materials**

Supplementary information S1. How DC-CART works.

Supplementary information S2. The mechanism of filter membrane obstruction and the method of filter membrane washing

Supplementary table S1. Characteristics of the study ascites before propensity score matching.

Supplementary table S2. Adverse events of patients receiving DC-CART using each filter.

**Supplementary information S1:** How DC-CART works.


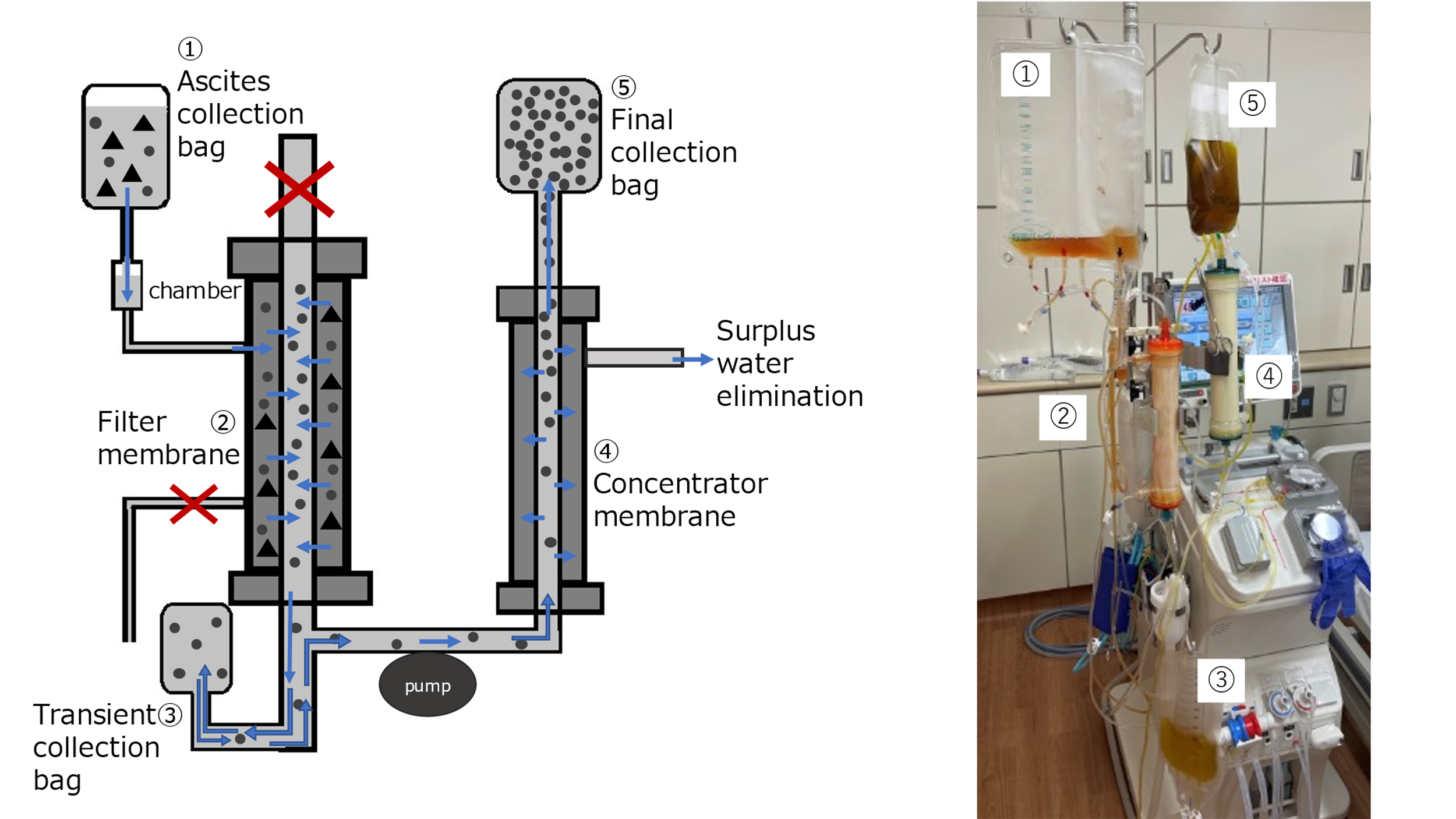


※①-⑤ in the above figures correspond to the following explanatory texts ①-⑤.

1. Ascites drained from the patient’s abdominal cavity is set here. A height difference pressure is required as the driving force for filtration in DC-CART procedure. In our hospital, the ascites collection bag hangs at height of 1.7 m. As can be seen in this figure, no pump is used in the filtration process. The ascites water flows toward the filter membrane using the height difference pressure.
2. Ascites passes through the filter membrane from the outside to the inside of hollow fibers. Bacteria, fibrins, and various cells including malignant cells, inflammatory cells, and blood cells are eliminated by the filter membrane. (Below shows the image of the surface of the filter membrane.)


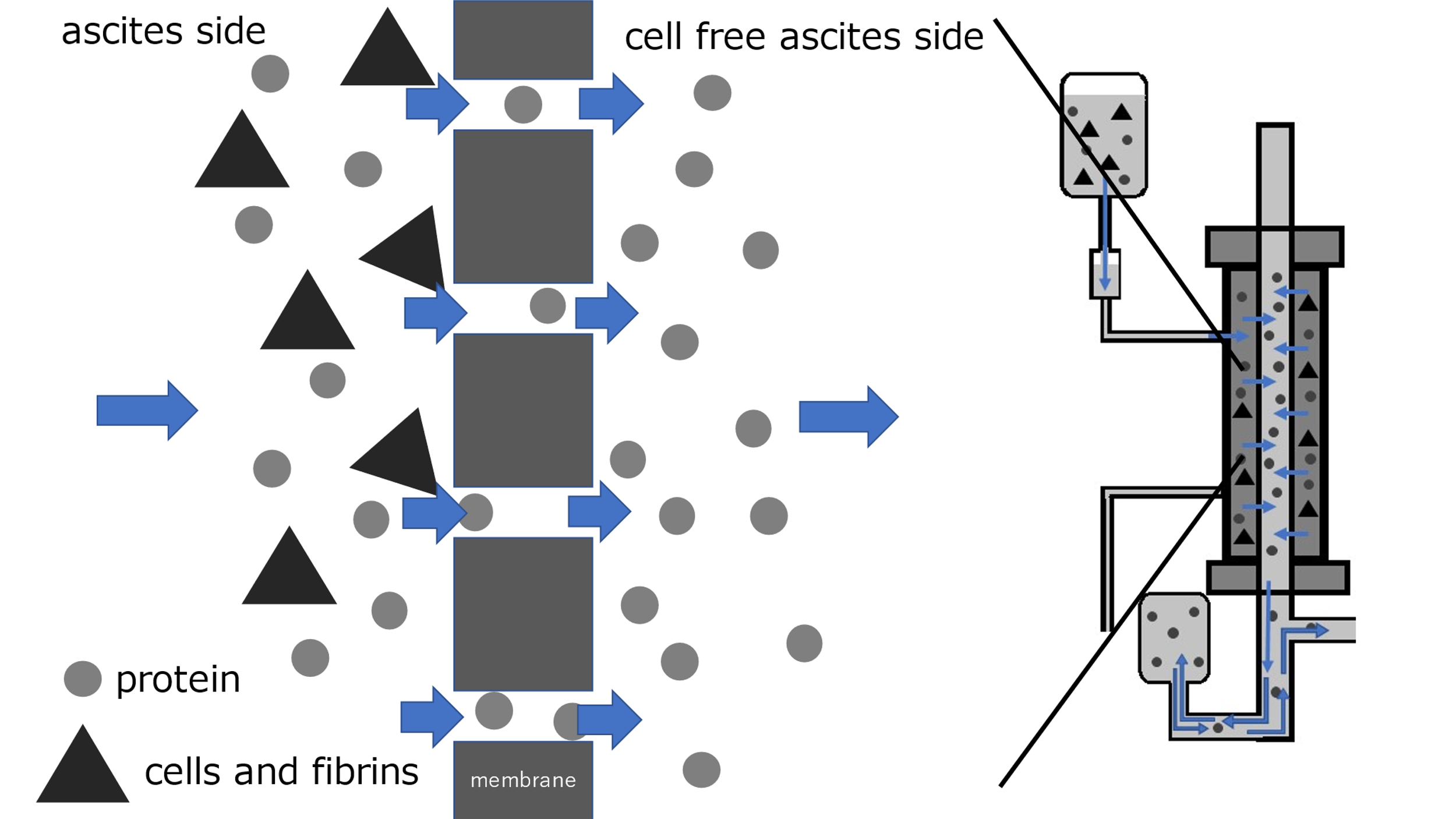


1. Filtered and purified ascites is kept in the transient collection bag.
2. The ascites in the transient bag is transferred to a concentrator membrane by a roller pump placed before the concentrator membrane. The concentrator membrane removes surplus water by ultrafiltration.
3. Concentrated ascites fluid was accumulated in the final collection bag, and the final product is administered intravenously into the patient's bloodstream.

**Supplementary information S2**: The mechanism of filter membrane obstruction and the method of filter membrane washing.

As explained in supplementary information S1, during the filtration process of ascites, cellular components accumulate on the outside surface of the filter membrane. The membrane surface is gradually covered with the accumulation, becomes clogged and obstructed, and the membrane is no longer capable of filtration. When the filter membrane is obstructed, the flow of ascites in the chamber stops. (This is “filter membrane obstruction”.)


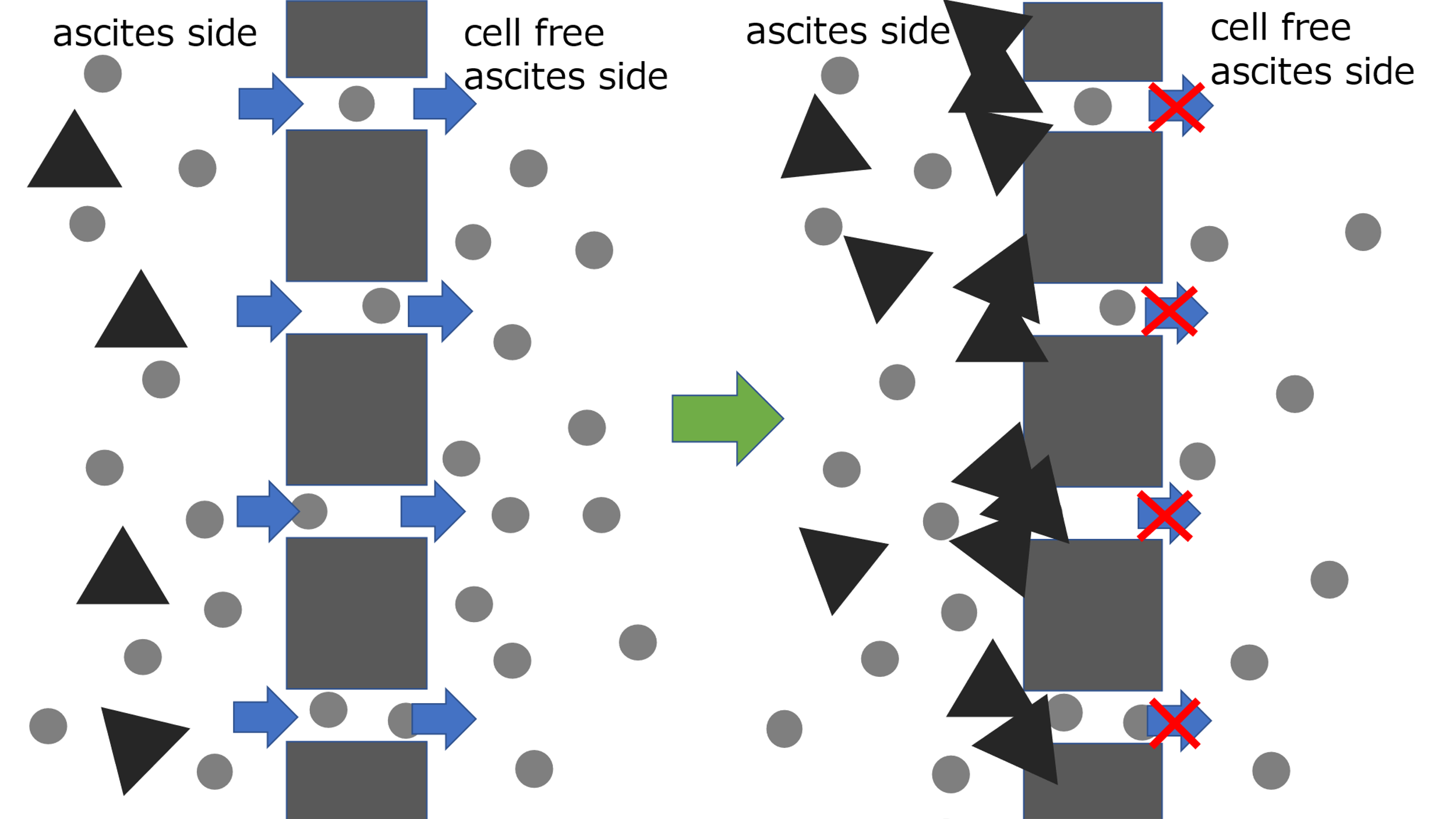


If the filter membrane obstruction occurred, it is cleaned with saline. As shown in the figure below, the filter membrane is washed by allowing saline to flow backward from the inside of the filter membrane ② to the outside. Set up the 500 mL saline bag ① and the washing solution bag ③. When the filter membrane becomes obstructed, the ascites inflow and outflow routes are clamped (X marks), a drainage route is opened, and then, saline is flushed into the system by height differential pressure. This process takes approximately 3 mins. Pressure washing with saline using a syringe is not performed because it is not recommended by the CART safety standards of the Japanese Society for Apheresis.
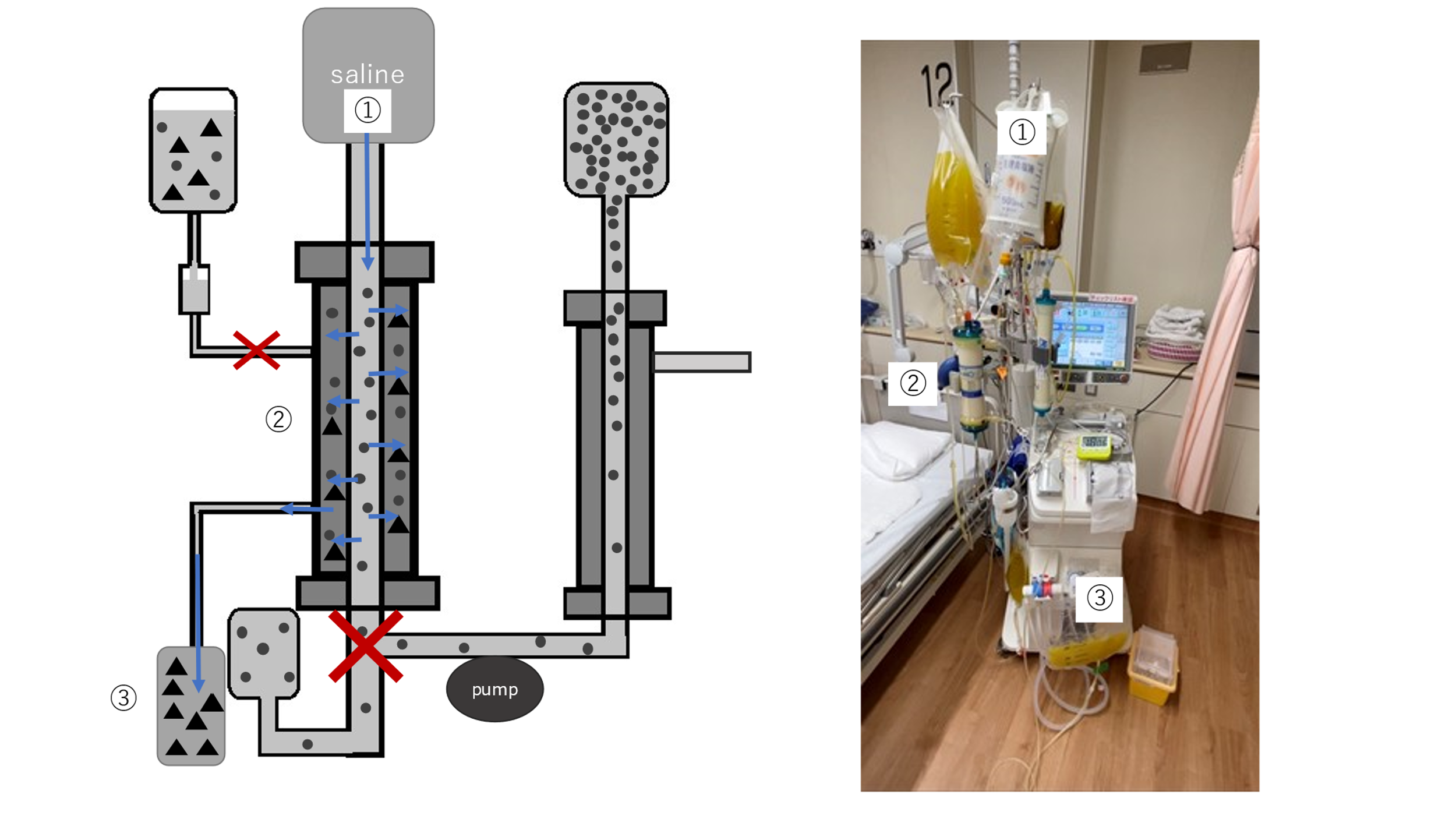


Below is an image of the filtration membrane surface when the filtration membrane washing process is performed. By this washing procedure, the accumulated fibrin and cell depositions are removed, and the ascites can be filtered again.


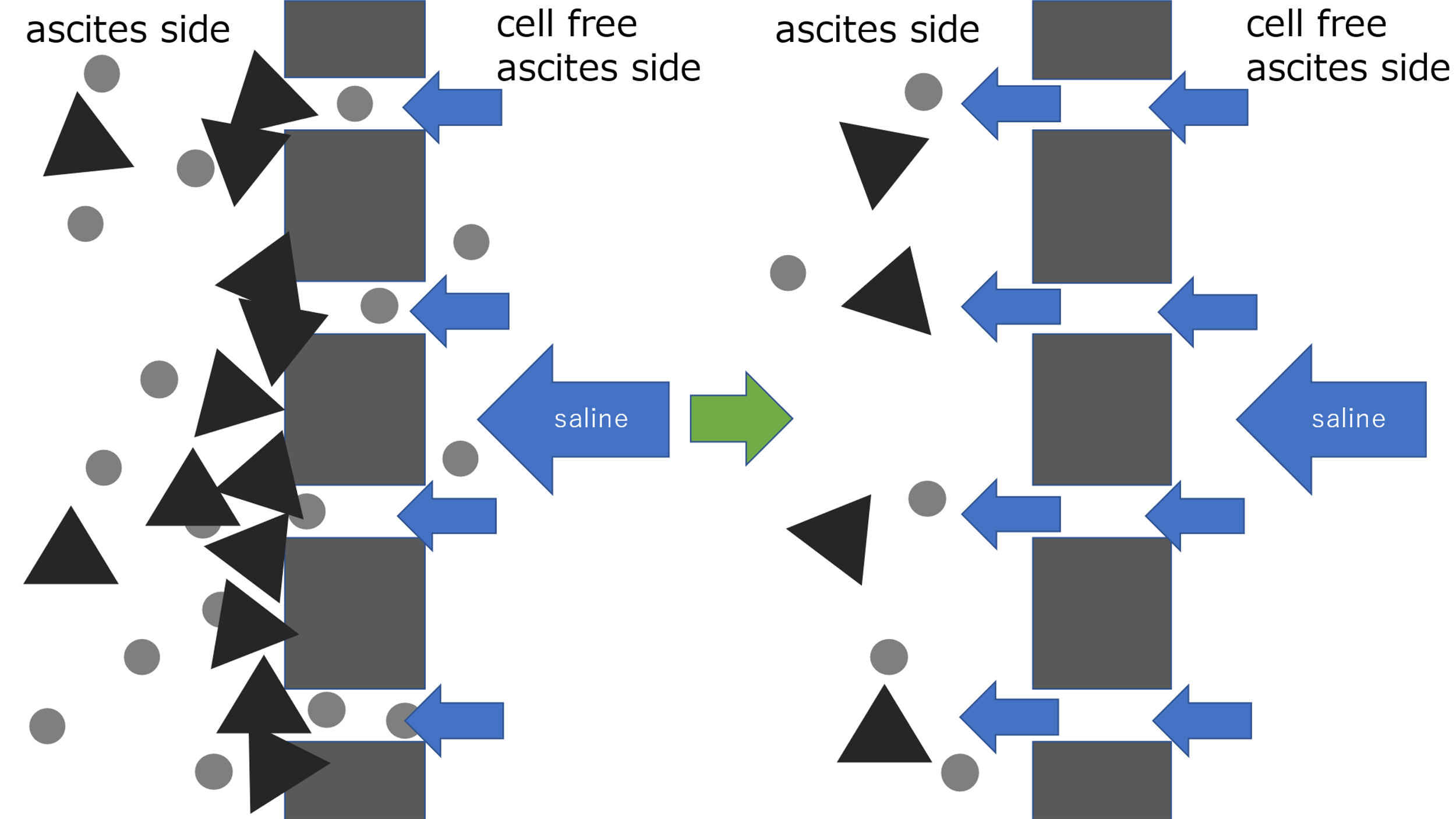


| Supplementary table S1. Characteristics of the study ascites before propensity score matching | | | |
| --- | --- | --- | --- |
|  | PE filter  N=99 | n-PES filter  N=72 | P-value |
| Volume of drained ascites (mL) | 5400 [3800-8700] | 4750 [3475-6225] | 0.035 |
| **Comorbidities** | | | |
| Liver cirrhosis | 64 (64.6%) | 39 (54.2%) | 0.206 |
| All cancer | 65 (65.7%) | 54 (75.0%) | 0.239 |
| gastric cancer | 13 (13.1%) | 2 (2.8%) | 0.026 |
| liver cancer | 21 (21.2%) | 17 (23.6%) | 0.714 |
| bile duct cancer | 2 (2.0%) | 14 (19.4%) | <0.001 |
| ovarian cancer | 13 (13.1%) | 3 (4.2%) | 0.062 |
| pancreatic cancer | 5 (5.1%) | 8 (11.1%) | 0.155 |
| other cancer | 11 (11.1%) | 10 (13.9%) | 0.641 |
| **Laboratory data** | | | |
| Ascitic total protein (g/dL) | 1.20 [0.90-2.50] | 1.90 [1.28-3.0] | 0.066 |
| Ascitic albumin (g/dL) | 0.60 [0.50-1.30] | 0.90 [0.50-1.20] | 0.966 |
| Ascitic total bilirubin (mg/dL) | 0.37 [0.21-0.56] | 0.26 [0.21-0.45] | 0.172 |
| Ascitic direct bilirubin (mg/dL) | 0.12 [0.07-0.22] | 0.10 [0.09-0.18] | 0.803 |
| Ascitic total cholesterol (mg/dL) | 16 [12-47] | 31 [18-40] | 0.037 |
| Ascitic lactate dehydrogenase (U/L) | 57 [43-280] | 61.5 [46.75-91.75] | 0.811 |
| Ascitic hyaluronic acid (ng/mL) | 4780 [3010-8800] | 5405 [3357.5-7662.5] | 0.811 |
| Number of cells in ascites(/µL) | 67 [31-229.5] | 175.5[102.5-267.5] | <0.001 |
| Number of monocyte in ascites (/µL) | 48 [25-111] | 147.0 [88.5-239.0] | <0.001 |
| Number of segmented cell in ascites(/µL) | 2 [0-14] | 5 [2-12.25] | 0.033 |
| Number of other cells in ascites(/µL) | 3 [0-19] | 3 [2-8] | 0.672 |
| Qualitative data, number (percentage); P-values were calculated by the chi-square test. Quantitative data, median (IQR); P-values were calculated by the Mann-Whitney U test. | | | |

| Supplementary table S2. Adverse events of patients receiving DC-CART using each filter. | | | |
| --- | --- | --- | --- |
|  | PE filter | n-PES filter | P-value |
| n | 31 | 31 |  |
| fever (%) | 5 (16.1) | 9 (29.0) | 0.363 |
| anemia (%) | 5 (16.1) | 7 (22.6) | 0.749 |
| stagger (%) | 0 (0.0) | 3 (9.7) | 0.238 |
| insomnia (%) | 0 (0.0) | 2 (6.5) | 0.492 |
| others (%) | 3 (9.7) | 3 (9.7) | 1.000 |

Fever occurred when the body temperature rose by 1 C° or more after the treatment and when the body temperature was 37.5 C° or more. Anemia was defined as when the hemoglobin level decreased by 1 g/dL and the hemoglobin level was 10 g/dL or less. Stagger and insomnia refer to the medical records after the procedure. Others include pollakiuria, chest discomfort, hepatic encephalopathy, decreased SpO2, abdominal fullness, and chills.

**Supplementary figure S1**: There were 119sessions of ascites due to cancer. 48sessions (24 in n-PES filter group; 24 in PE filter group) were included in 1:1 propensity score matched analysis. All factors of baseline characteristics of ascites were similar between the n-PES filter group and PE filter group after propensity score matching. The Kaplan-Meier curves show the non-obstructive filter survival rate for each group.


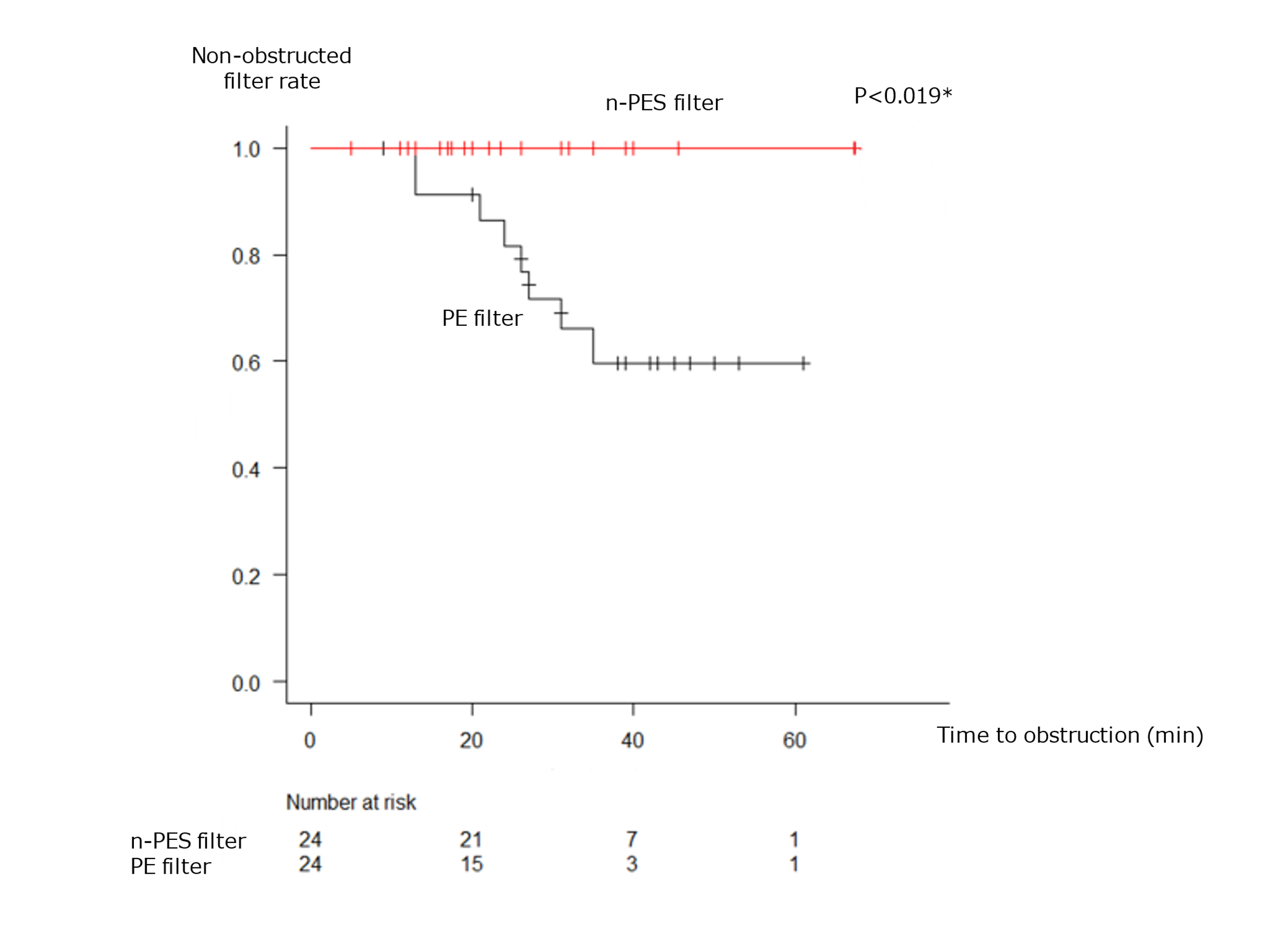


**Supplementary figure S2**: There were 103sessions of ascites due to liver cirrhosis. 24sessions (12 in n-PES filter group; 12 in PE filter group) were included in 1:1 propensity score matched analysis. All factors of baseline characteristics of ascites were similar between the n-PES filter group and PE filter group after propensity score matching. The Kaplan-Meier curves show the non-obstructive filter survival rate for each group.


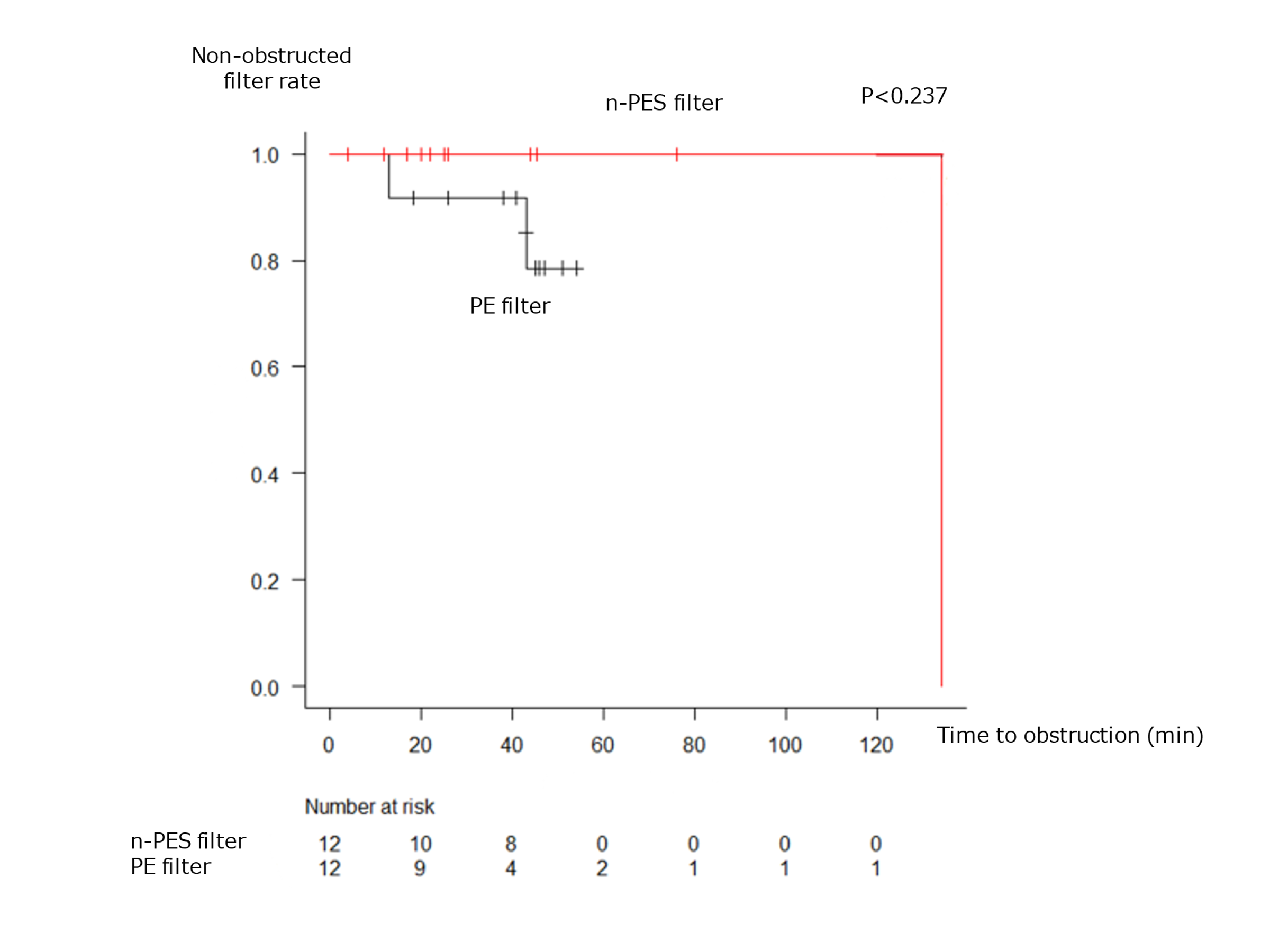

Supplement: Supplementary file 1 — Data S1. [file AOR-49-592-s001.docx]
